# The complete mitochondrial genome of *Pelecanus occidentalis* (Pelecaniformes: Pelecanidae) and its phylogenetic analysis

**DOI:** 10.1080/23802359.2018.1491337

**Published:** 2018-07-11

**Authors:** Tian Huang, Jiao Peng, Yunlin Zhao, Zhenggang Xu

**Affiliations:** a College of Bioscience and Biotechnology, Hunan Agricultural University, Changsha, China;; b College of Information and Electronic Engineering, Hunan City University, YiYang, Hunan Province, China;; c Hunan Engineering Research Center for Internet of Animals, Changsha, China;; d Key Laboratory of Forestry Remote Sensing Based Big Data & Ecological Security for Hunan Province, Central South University of Forestry and Technology, Changsha, China

**Keywords:** *Pelecanus occidentalis*, Pelecaniformes, mitochondrial genome, phylogeny

## Abstract

*Pelecanus occidentalis*, in the order Pelecaniformes, is one of the most abundant and widespread waterbird species in the coast of America. However, the phylogenetic relationships among Pelecaniformes, Suliformes, and Ciconiiformes remain unresolved, particularly in Pelecanidae and Ciconiidae. In this study, we first sequenced and described the complete mitochondrial genome and phylogeny of *P. occidentalis*. The whole genome of *P. occidentalis* was 17,315 bp in length, and contained 13 protein-coding genes, 21 transfer RNA genes, two ribosome RNA genes, and one non-coding control region. The overall base composition of the mitochondrial DNA was 30.1% for A, 23.7% for T, 31.5% for C, and 14.6% for G, with a GC content of 46.1%. A phylogenetic tree confirmed that *P. occidentalis* (Pelecaniformes) was sister to *C. boyciana* (Ciconiiformes), and Ardeidae and Threskiornithidae were both monophyletic group. This information will be useful in the current understanding of the phylogeny and evolution of Pelecaniformes.

*Pelecanus occidentalis*, in the order Pelecaniformes, is one of the most abundant and widespread waterbird species in the coast of America. This species is listed as of Least Concern (LC) on the IUCN Red List of Threatened Species (IUCN 2017). Despite this, genetic information of *P. occidentalis* is quite limited, which lead to confused and contentious phylogenetic relationships among Pelecaniformes, Suliformes, and Ciconiiformes (Zhang et al. 2012). Normally, the Ciconiiformes is restricted to included only the family Ciconiidae, and the Pelecaniformes consists of Pelecanidae, Ardeidae, Threskiornithidae, Balaenicipitidae, and Scopidae (Clements et al. 2017) (based on the recommendation of the North American Classification Committee). However, this taxonomy has been questioned by Gibb (Gibb et al. 2013) and Zhang (Zhang et al. 2012), particularly in Pelecanidae and Ciconiidae. Therefore, we sequenced the complete mitochondrial genome of *P. occidentalis* to enhance our understanding of the phylogeny and evolution of Pelecaniformes.

The specimen was collected from southeastern United States and stored at Hunan Engineering Research Center for Internet of Animals, China. The total mitochondrial DNA was extracted from the muscle tissue using a DNeasy Plant Mini Kit (Qiagen, Valencia, CA), and sequenced by using the Illumina Miseq Platform (Illumina, San Diego, CA). Adapters and low-quality reads were removed using the NGS QC Toolkit (Patel and Jain 2012). The genome was annotated using the MITOS WEB Server (Bernt et al. 2013). Each of the annotated peotein-coding genes (PCGs) were compared with published sequences of other vertebrate species using DOGMA (Wyman et al. 2004). Drawing a circular map of the mitochondria referred to Zhang’s method (Zhang et al. 2017) by using OGDRAW (Lohse et al. 2007). Phylogenetic tree of the relationships among Pelecaniformes and its related orders were presented using 13 shared PCGs among 18 species by Neighbour-joining (NJ) analyses using MEGA 7.0 software with 1000 bootstrap replication (Kumar et al. 2016). The complete mitochondrial genome of *P. occidentalis* was submitted to the NCBI database under the accession number MH041272.

The complete mitochondrial genome of *P. occidentalis* was 17,315 bp in length. A total of 36 mitochondrial genes were identified, including 13 protein-coding genes (PCGs), 21 transfer RNA (tRNA) genes, two ribosomal RNA (rRNA) genes, and one non-coding control region (D-loop). Among these genes, *ND6* and eight tRNAs (*trn^Gln^*, *trn^Ala^*, *trn^Asn^*, *trn^Cys^*, *trn^Tyr^*, *trn^Ser^*, *trn^Pro^*, and *trn^Glu^*) were located on the light strand (L-strand), while all of the remaining genes were located on the heavy strand (H-strand). The overall base composition of *P. occidentalis* mitogenome was 30.1% for A, 23.7% for T, 31.5% for C, and 14.6% for G, with a GC content of 46.1%. The reconstructed phylogenetic tree supported the placement of *P. occidentalis* in Pelecaniformes (Ardeidae, Pelecanidae, Phalacrocoracidae, and Threskiornithidae), Suliformes (Sulidae), and Ciconiiformes (Ciconiidae) clade (Figure 1). All of the nodes were inferred with strong support by the NJ analysis. Our results confirmed that *P. occidentalis* (Pelecaniformes) was sister to *C. boyciana* (Ciconiiformes), and Ardeidae and Threskiornithidae were both monophyletic group. In all, the mitochondrial genome reported here would be useful in the current understanding of the phylogeny and evolution of Pelecaniformes.

**Figure 1.**
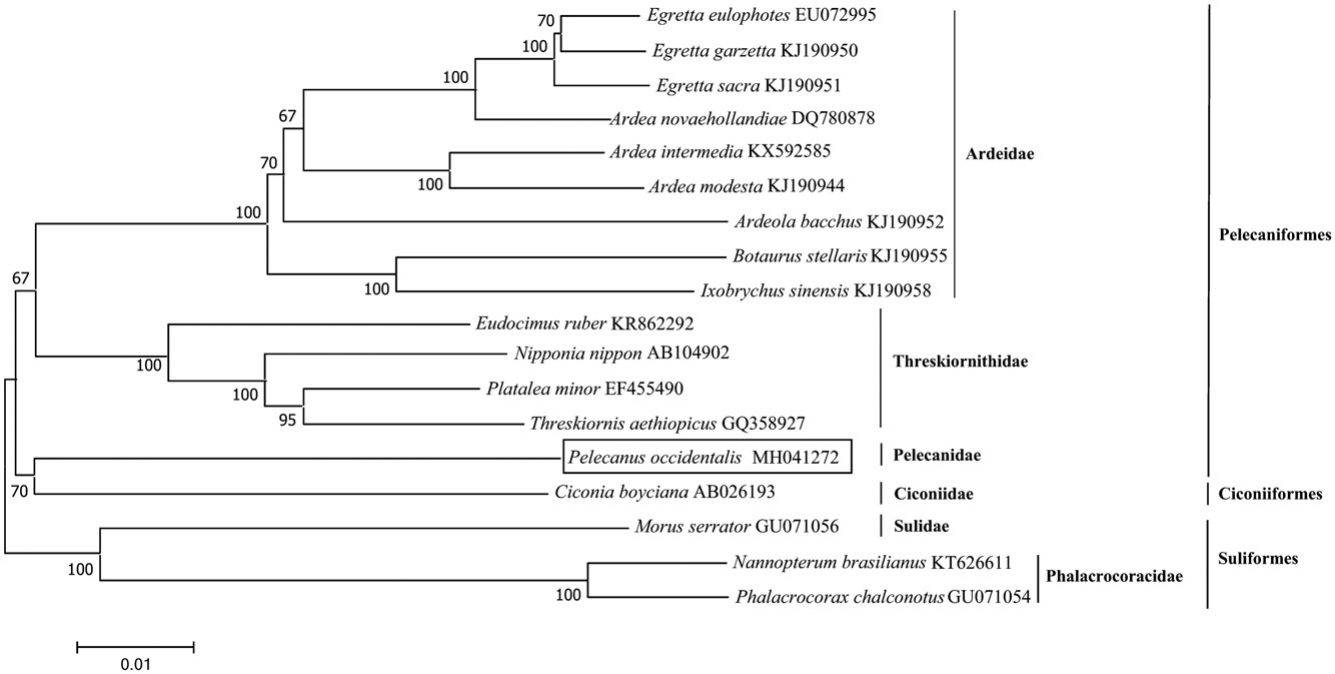
Phylogenetic tree of the relationships among Pelecaniformes and its related orders based on 13 PCGs. Branch lengths and topologies came from the Neighbour-joining (NJ) analyses.
